# Factor analysis based on SHapley Additive exPlanations for sepsis-associated encephalopathy in ICU mortality prediction using XGBoost — a retrospective study based on two large database

**DOI:** 10.3389/fneur.2023.1290117

**Published:** 2023-12-14

**Authors:** Jiayu Guo, Hongtao Cheng, Zicheng Wang, Mengmeng Qiao, Jing Li, Jun Lyu

**Affiliations:** ^1^Department of Clinical Research, The First Affiliated Hospital of Jinan University, Guangzhou, China; ^2^School of Public Health, Shannxi University of Chinese Medicine, Xianyang, China; ^3^School of Nursing, Jinan University, Guangzhou, Guangdong, China; ^4^Guangdong Provincial Key Laboratory of Traditional Chinese Medicine Informatization, Guangzhou, Guangdong, China

**Keywords:** sepsis-associated encephalopathy (SAE), XGBoost, SHAP (SHapley Additive exPlanations), ICU mortality, eICU-CRD, MIMIC-IV

## Abstract

**Objective:**

Sepsis-associated encephalopathy (SAE) is strongly linked to a high mortality risk, and frequently occurs in conjunction with the acute and late phases of sepsis. The objective of this study was to construct and verify a predictive model for mortality in ICU-dwelling patients with SAE.

**Methods:**

The study selected 7,576 patients with SAE from the MIMIC-IV database according to the inclusion criteria and randomly divided them into training (*n* = 5,303, 70%) and internal validation (*n* = 2,273, 30%) sets. According to the same criteria, 1,573 patients from the eICU-CRD database were included as an external test set. Independent risk factors for ICU mortality were identified using Extreme Gradient Boosting (XGBoost) software, and prediction models were constructed and verified using the validation set. The receiver operating characteristic (ROC) and the area under the ROC curve (AUC) were used to evaluate the discrimination ability of the model. The SHapley Additive exPlanations (SHAP) approach was applied to determine the Shapley values for specific patients, account for the effects of factors attributed to the model, and examine how specific traits affect the output of the model.

**Results:**

The survival rate of patients with SAE in the MIMIC-IV database was 88.6% and that of 1,573 patients in the eICU-CRD database was 89.1%. The ROC of the XGBoost model indicated good discrimination. The AUCs for the training, test, and validation sets were 0.908, 0.898, and 0.778, respectively. The impact of each parameter on the XGBoost model was depicted using a SHAP plot, covering both positive (acute physiology score III, vasopressin, age, red blood cell distribution width, partial thromboplastin time, and norepinephrine) and negative (Glasgow Coma Scale) ones.

**Conclusion:**

A prediction model developed using XGBoost can accurately predict the ICU mortality of patients with SAE. The SHAP approach can enhance the interpretability of the machine-learning model and support clinical decision-making.

## Introduction

Sepsis, a syndrome caused by dysfunction of organs including the central nervous system (CNS), heart, and lungs (1–3) due to dysregulation of the host response to infection, is the most common cause of death in intensive care unit patients worldwide. One manifestation of sepsis-induced cerebral dysfunction is sepsis-associated encephalopathy (SAE), which is defined as diffuse cerebral dysfunction secondary to organic infection in the absence of an obvious central nervous system infection (4).

The pathophysiology of SAE is intricate, arising from a convergence of inflammatory and non-inflammatory processes impacting various categories of cerebral cells. Significant mechanisms encompass heightened microglial activation, disruption of the blood–brain barrier (BBB), and the perpetuation of an extended inflammatory reaction (5). Upon the initial emergence of sepsis, an inordinate immune-inflammatory response is incited, setting in motion the infiltration of inflammatory mediators into cerebral tissue, thereby activating microglial cells. This activation gives rise to the establishment of a cytotoxic milieu, instigating the release of reactive oxygen species, nitric oxide (6), and glutamate, as a countermeasure against sepsis. Nevertheless, the CNS is notably vulnerable to neurotoxic agents such as free radicals, inflammatory mediators, and intravascular proteins, thus precipitating a malfunction in the BBB (7). The relentless activation of microglia perpetuates a deleterious cycle, culminating in aberrant neuronal performance and cellular demise, thereby exacerbating BBB impairment and the progression of SAE. In addition to this, sepsis damages the hippocampus, cortex, cerebellum and brainstem of the brain. Sepsis-driven brain damage occurs in a diffuse form and is strongly associated with cognitive impairment.

Clinicians must exclude primary CNS disorders, sedation-related cognitive disorders, metabolic encephalopathies, and poisonings before diagnosing SAE on the basis of cognitive and neuropsychiatric deficits, manifestations of delirium, or a Glasgow Coma Scale (GCS) score of less than 15 (8). Globally, up to 50% of intensive care unit (ICU) patients present with SAE during sepsis (4, 9), which tends to increase the length of stay and mortality of septic patients in the ICU (10). The current lack of specific treatment options and insufficient understanding of the underlying mechanisms of SAE are the most common causes of poor prognosis in sepsis. Therefore, the aim of this study was to investigate the independent risk factors for ICU death in patients with SAE and to develop a predictive model to quantify the likelihood of ICU death in patients with SAE.

## Materials and methods

### Data source

Data for this study were obtained from the MIMIC-IV and eICU-CRD databases, with the former being a multiparametric, structured single-center critical-care database published in 2003 that includes clinically available data on more than 380,000 patients during 2008–2019. There was no requirement to obtain permission from individual patients or ethical approval statements because the initiative had no impact on clinical care and none of the patients in the database could be identified (11). Our study also followed the guidelines of the Declaration of Helsinki and Transparent Reporting of a Multivariate Prediction Model for Individual Prognosis or Diagnosis (12).

The eICU-CRD database contains data from the ICU wards of numerous hospitals in the US. It contains routine data on 200,859 patients obtained from more than 300 hospitals in the US during 2014 and 2015 (13). No specific patient permission was needed because both databases use anonymous health data.

### Patient population

Presently, there exists a deficiency of precise diagnostic modalities for SAE. Clinical diagnosis relies on exclusion and necessitates discrimination from central nervous system infections, metabolic encephalopathy (a widespread yet potentially reversible cerebral dysfunction arising from metabolic or toxic origins), excessive sedative ingestion, and withdrawal manifestations with the potential to impact sensory faculties.

**Figure 1:**
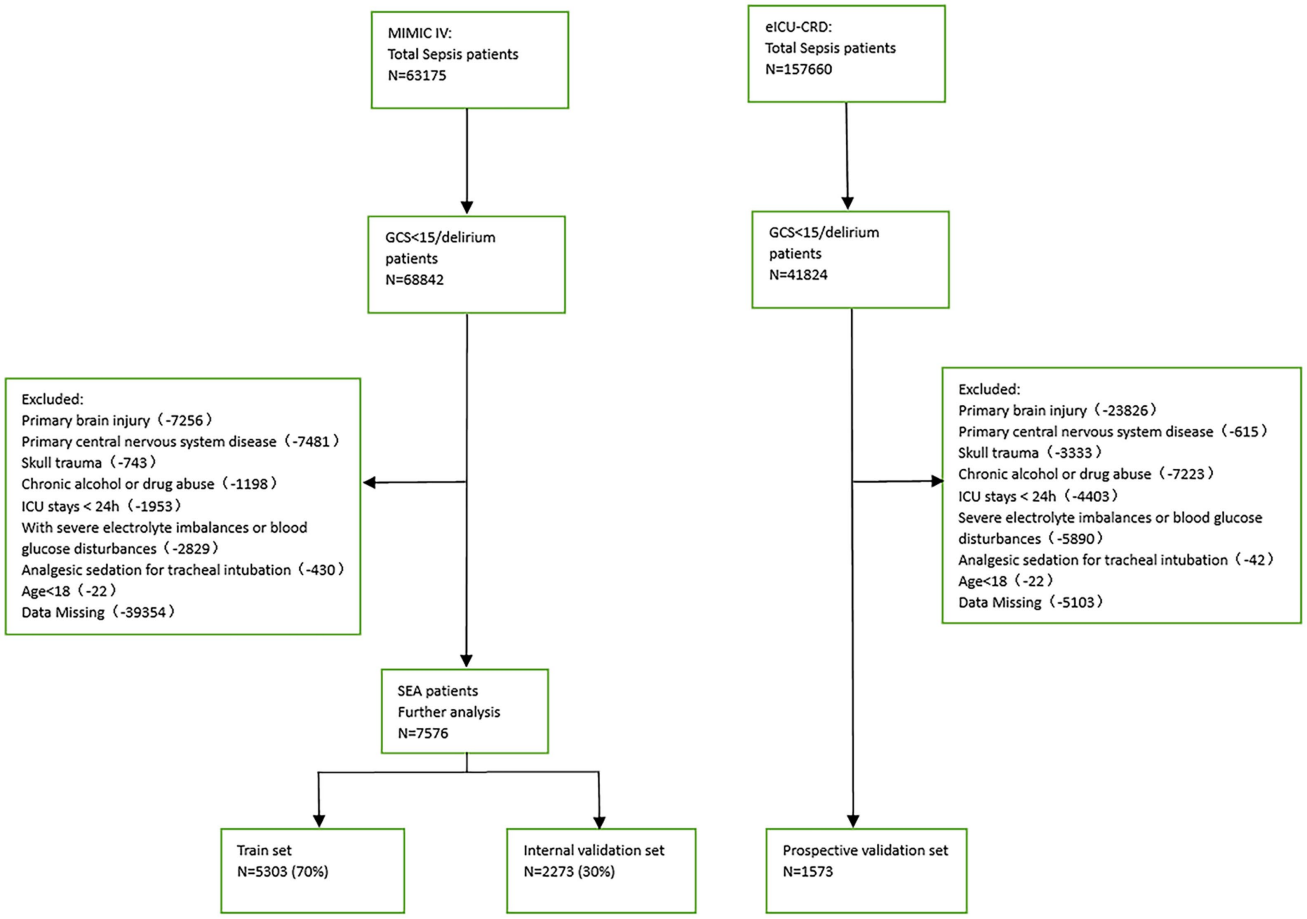
Flowchart of patient cohorts.

Patients with a Sequential Organ Failure Assessment (SOFA) score ≥ 2 based on the Sepsis-3 classification and a GCS score < 15 or delirium on the day before admission to the ICU were considered SAE patients. The exclusion criteria were (1) presence of primary brain injury, (2) psychiatric disorders and neurological diseases, (3) metabolic, hepatic, hypertensive, or toxic encephalopathy, (4) severe electrolyte disturbance or deglycation, (5) patients who were intubated, given analgesics, and sedated at the time of admission, (6) long-term alcohol or drug abuse, or (7) an ICU stay of <24 h. Figure 1 depicts the flow chart for case inclusion.

### Observation indicators

This study used Structured Query Language to extract the following basic information of patients from both databases: age, gender, and mean values of vital signs at the time of first ICU admission, including heart rate, respiratory rate, and body temperature. From the time of ICU admission, the first laboratory data included ghrelin, lymphocytes, eosinophils, neutrophils, monocytes, hemoglobin, urea nitrogen, platelets, creatinine, glucose, blood urea nitrogen (BUN), hematocrit (HCT), partial thromboplastin time (PTT), white blood cell count (WBC), normalized ratio (INR), anion gap (AG), mean corpuscular hemoglobin (MCH), mean corpuscular hemoglobin concentration (MCHC), Mean Corpuscular Volume (MCV) and red blood cell distribution width (RDW), Severity was determined using the acute physiology score III (APSIII), SOFA score, GCS score, and comorbidity occurrence.

### Statistical analysis

Statistical processing was performed using R software (version 4.2.2). For continuous variables, values are expressed as standard deviation or median of interquartile range (IQR); for categorical variables, values are expressed as total (%). Comparisons of continuous variables were made using the *t* test or Wilcoxon rank sum test, and comparisons of proportions were made using the χ 2 test or Fisher exact test. After the variables were identified by Extreme Gradient Boosting (XGBoost), we used these included clinical and laboratory variables to construct a prediction model for in-ICU mortality in SAE patients based on the XGBoost algorithm. XGBoost is an improved algorithm based on gradient boosting decision trees that efficiently constructs boosted trees and runs them in parallel (14). The core of the algorithm is to optimize the value of the objective function (15). In model development and comparison, we use a 5-fold cross-validation approach, which provides a more stable and reliable way to measure the performance of the model.

The prediction model was trained and internally validated using training and test sets randomized at a ratio of 7:3. The performance of the prediction model was externally validated using the identical data of patients with SAE from the eICU-CRD database. The predictive values of different models were analyzed using the receiver operating characteristic (ROC) and area under the ROC curve (AUC), with the latter allowing quantitative differentiation of column line graphs. In the XGBoost analysis, qualitative data were converted to numerical data, and “yes” and “no” were converted to “1” and “0,” respectively.

An aesthetically pleasing additional interpretation method, the SHapley Additive exPlanations (SHAP), was used in XGBoost to increase the readability of the model. SHAP is a technique used to explain the output of any machine-learning model (16). A SHAP summary plot was used to present the effect of the characteristics attributed to the model. Colors in the scatter plot intuitively represent the correlation between the characteristic value and the anticipated probability. The importance of specific features and their impacts on the output of the model were examined using the SHAP dependence plot. A SHAP force plot was used to illustrate how important characteristics affect use of the final model across all patients.

## Results

### Baseline patient characteristics

This study used 1,573 patients from the eICU-CRD database as the external test set, and 7,576 patients from the MIMIC-IV database were randomly split into a training (*n* = 5,303, 70%) and an internal test (*n* = 2,273, 30%) set.

Among the 7,576 patients within the MIMIC-IV database afflicted by SAE, 6709 (88.6%) experienced survival, while 867 (11.4%) succumbed prior to ICU discharge. In the case of the 1,573 patients drawn from the eICU-CRD database, 1402 (89.1%) survived, and 171 (10.9%) met their demise. Across the training, internal test, and external test sets, the majority of patients were aged over 65 years (with mean ages of 69, 70, and 66 years, respectively) and were predominantly male (comprising 59.2, 59.6, and 57.8% of the respective cohorts). The average length of ICU stay was 3.38 days for the training set, 3.29 days for the internal test set, and 3.99 days for the external test set. Additionally, many of these patients presented with comorbidities, including congestive heart failure (35.9, 36.6, and 7.3%), COPD (28.1, 25.9, and 1.1%), diabetes (9.4, 9.2, and 0.8%), renal failure (25.8, 25.6, and 9.4%), or liver disease (11.5, 13.0, and 1.4%).

The demographic profiles and fundamental patient information pertaining to the training and test sets are delineated in Tables 1, 2. In Table 3, we present the foundational attributes of the study cohort sourced from the MIMIC-IV database, stratified by distinct outcomes. Notably, the average age of patients afflicted by SAE was notably higher in the deceased group in comparison to the survivor group. Furthermore, the incidence rates of myocardial infarction, peripheral vascular disease, dementia, diabetes, sclerosis, and liver disease exhibited variations between these two cohorts. Conversely, no statistically significant disparities were observed between the two groups concerning myocardial infarction, peripheral vascular disease, dementia, diabetes, sodium levels, and MCH. Turning our attention to the baseline attributes of the subjects derived from the eICU-CRD database for different outcomes, these details are summarized in Table 4. Significant differences among groups were discerned in variables such as sedative usage, analgesic administration, vasopressin and norepinephrine dosages, GCS score, SOFA score, lactate levels, creatinine values, bicarbonate levels, BUN, PTT, INR, AG, MCHC, RDW, respiratory rate, and body temperature.

**Table 1:** Research subject base information form (internal validation).

	Total	Training cohorts	Internal validation cohorts	*p* value
**N**	7,576	5,303	2,273	
Death = No/Yes (%)	6709/867 (88.6/11.4)	4690/613 (88.4/11.6)	2019/254 (88.8/11.2)	0.658
ICU stay time, days (median [IQR])	3.33 [2.00, 6.29]	3.38 [2.00, 6.33]	3.29 [1.96, 6.13]	0.363
Age (median [IQR])	70.00 [58.00, 79.00]	69.00 [58.00, 79.00]	70.00 [59.00, 79.00]	0.706
Gender = Male/Female (%)	4497/3079 (59.4/40.6)	3142/2161 (59.2/40.8)	1355/918 (59.6/40.4)	0.787
**Medical treatments, *n* (%)**				
Sedatives = No/Yes	1292/6284 (17.1/82.9)	900/4403 (17.0/83.0)	392/1881 (17.2/82.8)	0.797
Analgesic = No/Yes	729/6847 (9.6/90.4)	492/4811 (9.3/90.7)	237/2036 (10.4/89.6)	0.131
Antibiotic = No/Yes	988/6588 (13.0/87.0)	689/4614 (13.0/87.0)	299/1974 (13.2/86.8)	0.877
Vasopressin = No/Yes	6642/934 (87.7/12.3)	4634/669 (87.4/12.6)	2008/265 (88.3/11.7)	0.262
**Comorbidity, *n* (%)**				
Myocardial infarct = No/Yes	5997/1579 (79.2/20.8)	4193/1110 (79.1/20.9)	1804/469 (79.4/20.6)	0.793
Congestive heart failure = No/Yes	4837/2739 (63.8/36.2)	3397/1906 (64.1/35.9)	1440/833 (63.4/36.6)	0.576
Peripheral vascular disease = No/Yes	6397/1179 (84.4/15.6)	4468/835 (84.3/15.7)	1929/344 (84.9/15.1)	0.523
Dementia = No/Yes	7353/223 (97.1/2.9)	5151/152 (97.1/2.9)	2202/71 (96.9/3.1)	0.594
COPD = No/Yes	5499/2077 (72.6/27.4)	3814/1489 (71.9/28.1)	1685/588 (74.1/25.9)	0.051
Liver disease = No/Yes	6671/905 (88.1/11.9)	4694/609 (88.5/11.5)	1977/296 (87.0/13.0)	0.064
Diabetes = No/Yes	6871/705 (90.7/9.3)	4807/496 (90.6/9.4)	2064/209 (90.8/9.2)	0.862
Renal disease = No/Yes	5627/1949 (74.3/25.7)	3935/1368 (74.2/25.8)	1692/581 (74.4/25.6)	0.852
**Severe score, median (IQR)**				
SOFA	7.00 [5.00, 10.00]	7.00 [5.00, 9.00]	7.00 [5.00, 10.00]	0.12
GCS	13.00 [8.00, 14.00]	13.00 [8.00, 14.00]	13.00 [8.00, 14.00]	0.054
APSIII	54.00 [39.00, 75.00]	54.00 [39.00, 75.00]	54.00 [39.00, 77.00]	0.298
**Laboratory tests, median (IQR)**				
Lactate (mmol/L)	1.80 [1.30, 2.60]	1.80 [1.30, 2.60]	1.80 [1.30, 2.55]	0.4
Glucose (mg/dl)	128.50 [113.50, 144.78]	128.25 [113.00, 145.07]	129.10 [114.94, 144.25]	0.303
Creatinine (mg/dl)	1.05 [0.75, 1.65]	1.05 [0.75, 1.65]	1.05 [0.75, 1.65]	0.854
BUN (K/uL)	21.00 [14.50, 35.50]	21.00 [14.50, 35.00]	21.50 [14.50, 37.00]	0.064
Platelets (K/ul)	178.50 [129.50, 248.00]	180.00 [130.00, 249.00]	176.50 [127.50, 246.00]	0.128
Potassium (K/ul)	4.25 [3.90, 4.65]	4.25 [3.90, 4.65]	4.25 [3.95, 4.65]	0.045
Sodium (K/ul)	138.50 [136.00, 141.00]	138.50 [136.00, 141.00]	138.50 [136.00, 141.00]	0.539
Bicarbonate (meq/L)	23.00 [20.50, 25.50]	23.00 [20.50, 25.50]	23.00 [20.50, 25.00]	0.767
Calcium (mg/dl)	8.20 [7.80, 8.70]	8.20 [7.80, 8.70]	8.20 [7.80, 8.65]	0.93
Chloride (mmol/L)	105.00 [101.00, 108.00]	105.00 [101.00, 108.00]	105.00 [101.00, 108.00]	0.416
HCT (%)	10.10 [8.90, 11.55]	10.10 [8.85, 11.50]	10.15 [8.95, 11.65]	0.143
PTT (s)	32.55 [28.05, 41.66]	32.60 [28.10, 41.90]	32.50 [28.00, 40.90]	0.329
WBC (K/uL)	12.10 [8.85, 16.05]	12.10 [8.85, 16.10]	12.00 [8.80, 15.90]	0.529
INR	1.30 [1.15, 1.55]	1.30 [1.15, 1.55]	1.30 [1.15, 1.55]	0.938
AG	14.00 [12.00, 16.50]	14.00 [12.00, 16.50]	13.50 [11.50, 16.50]	0.654
MCH (pg)	30.00 [28.60, 31.30]	30.00 [28.60, 31.30]	30.10 [28.60, 31.30]	0.783
MCHC (g/dL)	32.80 [31.60, 33.90]	32.80 [31.60, 33.90]	32.80 [31.70, 33.80]	0.899
MCV (fL)	91.00 [87.00, 95.00]	91.00 [87.00, 95.00]	91.00 [87.00, 95.00]	0.704
RDW (%)	15.00 [13.80, 16.70]	15.10 [13.80, 16.70]	15.00 [13.90, 16.50]	0.324
**Vital signs, median (IQR)**				
Heartrate (min^−1^)	104.00 [90.00, 119.00]	104.00 [90.00, 119.00]	104.00 [91.00, 120.00]	0.071
Respiratory rate (min^−1^)	27.00 [23.50, 32.00]	27.00 [23.00, 32.00]	28.00 [24.00, 32.00]	0.033
Temperature (°C)	36.33 [35.61, 37.33]	36.33 [35.61, 37.33]	36.33 [35.61, 37.33]	0.555

**Table 2:** Research subject base information form (external validation).

	Total	Training cohorts	External validation cohorts	*p* value
**N**	6876	5,303	1,573	
Death = No/Yes (%)	6092/784 (88.6/11.4)	4690/613 (88.4/11.6)	1402/171 (89.1/10.9)	0.478
ICU stay time, days (median [IQR])	3.54 [2.04, 6.71]	3.38 [2.00, 6.33]	3.99 [2.28, 7.49]	<0.001
Age (median [IQR])	69.00 [58.00, 78.00]	69.00 [58.00, 79.00]	66.00 [55.00, 76.00]	<0.001
Gender = Male/Female (%)	4051/2825 (58.9/41.1)	3142/2161 (59.2/40.8)	909/664 (57.8/42.2)	0.315
**Medical treatments, *n* (%)**				
Sedatives = No/Yes	2093/4783 (30.4/69.6)	900/4403 (17.0/83.0)	1193/380 (75.8/24.2)	<0.001
Analgesic = No/Yes	1867/5009 (27.2/72.8)	492/4811 (9.3/90.7)	1375/198 (87.4/12.6)	<0.001
Antibiotic = No/Yes	2261/4615 (32.9/67.1)	689/4614 (13.0/87.0)	1572/1 (99.9/0.1)	<0.001
Vasopressin = No/Yes	6061/815 (88.1/11.9)	4634/669 (87.4/12.6)	1427/146 (90.7/9.3)	<0.001
**Comorbidity, *n* (%)**				
Myocardial infarct = No/Yes	5709/1167 (83.0/17.0)	4193/1110 (79.1/20.9)	1516/57 (96.4/3.6)	<0.001
Congestive heart failure = No/Yes	4855/2021 (70.6/29.4)	3397/1906 (64.1/35.9)	1458/115 (92.7/7.3)	<0.001
Peripheral vascular disease = No/Yes	6029/847 (87.7/12.3)	4468/835 (84.3/15.7)	1561/12 (99.2/0.8)	<0.001
Dementia = No/Yes	6710/166 (97.6/2.4)	5151/152 (97.1/2.9)	1559/14 (99.1/0.9)	<0.001
COPD = No/Yes	5370/1506 (78.1/21.9)	3814/1489 (71.9/28.1)	1556/17 (98.9/1.1)	<0.001
Liver disease = No/Yes	6245/631 (90.8/9.2)	4694/609 (88.5/11.5)	1551/22 (98.6/1.4)	<0.001
Diabetes = No/Yes	6368/508 (92.6/7.4)	4807/496 (90.6/9.4)	1561/12 (99.2/0.8)	<0.001
Renal disease = No/Yes	5360/1516 (78.0/22.0)	3935/1368 (74.2/25.8)	1425/148 (90.6/9.4)	<0.001
**Severe score, median (IQR)**				
SOFA	7.00 [5.00, 9.00]	7.00 [5.00, 9.00]	7.00 [5.00, 9.00]	0.043
GCS	13.00 [8.00, 14.00]	13.00 [8.00, 14.00]	11.00 [7.00, 14.00]	<0.001
APSIII	55.00 [40.00, 75.00]	54.00 [39.00, 75.00]	56.00 [42.00, 77.00]	<0.001
**Laboratory tests, median (IQR)**				
Lactate (mmol/L)	1.80 [1.20, 2.60]	1.80 [1.30, 2.60]	1.70 [1.10, 2.80]	0.011
Glucose (mg/dl)	127.75 [111.00, 145.29]	128.25 [113.00, 145.07]	124.00 [105.00, 146.00]	<0.001
Creatinine (mg/dl)	1.10 [0.79, 1.70]	1.05 [0.75, 1.65]	1.18 [0.82, 1.89]	<0.001
BUN (K/uL)	21.50 [14.50, 35.50]	21.00 [14.50, 35.00]	23.00 [14.00, 37.00]	0.068
Platelets (K/ul)	177.00 [126.50, 246.00]	180.00 [130.00, 249.00]	167.00 [117.00, 236.00]	<0.001
Potassium (K/ul)	4.20 [3.85, 4.60]	4.25 [3.90, 4.65]	4.10 [3.60, 4.51]	<0.001
Sodium (K/ul)	138.50 [136.00, 141.00]	138.50 [136.00, 141.00]	139.00 [136.00, 142.00]	0.029
Bicarbonate (meq/L)	23.00 [20.00, 25.50]	23.00 [20.50, 25.50]	23.00 [20.00, 25.00]	0.006
Calcium (mg/dl)	8.20 [7.70, 8.65]	8.20 [7.80, 8.70]	8.00 [7.40, 8.60]	<0.001
Chloride (mmol/L)	105.00 [101.00, 108.50]	105.00 [101.00, 108.00]	106.00 [102.00, 110.00]	<0.001
HCT (%)	10.90 [9.25, 14.40]	10.10 [8.85, 11.50]	30.60 [26.00, 35.90]	<0.001
PTT (s)	32.70 [28.10, 41.25]	32.60 [28.10, 41.90]	33.00 [28.20, 40.00]	0.582
WBC (K/uL)	12.10 [8.70, 16.35]	12.10 [8.85, 16.10]	12.00 [8.14, 17.10]	0.363
INR	1.30 [1.15, 1.55]	1.30 [1.15, 1.55]	1.30 [1.10, 1.60]	0.383
AG	13.50 [11.00, 16.00]	14.00 [12.00, 16.50]	11.00 [8.00, 15.00]	<0.001
MCH (pg)	30.00 [28.50, 31.20]	30.00 [28.60, 31.30]	29.80 [28.10, 31.10]	<0.001
MCHC (g/dL)	32.80 [31.70, 33.82]	32.80 [31.60, 33.90]	32.90 [31.90, 33.80]	0.227
MCV (fL)	91.00 [87.00, 95.00]	91.00 [87.00, 95.00]	90.00 [86.00, 94.60]	<0.001
RDW (%)	15.10 [13.90, 16.80]	15.10 [13.80, 16.70]	15.30 [14.00, 17.10]	<0.001
**Vital signs, median (IQR)**				
Heartrate (min^−1^)	101.00 [84.00, 117.00]	104.00 [90.00, 119.00]	92.00 [79.00, 109.00]	<0.001
Respiratory rate (min^−1^)	26.00 [18.00, 31.00]	27.00 [23.00, 32.00]	19.00 [15.00, 24.00]	<0.001
Temperature (°C)	36.39 [35.78, 37.28]	36.33 [35.61, 37.33]	36.70 [36.20, 37.10]	<0.001

**Table 3:** Comparison of basic characteristics of the surviving and dead groups in the MIMIC-IV database.

Variable	Survival	Death	*p* value
**Total**	6709	867	
ICU stay time, days, median (IQR)	3.2 (1.9,5.8)	5.3 (2.6,10.6)	<0.001
Age, year, median (IQR)	69 (58,79)	74 (64,82)	<0.001
Gender, male, *n* (%)	4014 (59.8)	483 (55.7)	0.02
**Medical treatments, *n* (%)**			
Sedatives	5502 (82)	782 (90.2)	<0.001
Analgesic	6178 (92.1)	669 (77.2)	<0.001
Antibiotic	5790 (86.3)	798 (92)	<0.001
Vasopressin	564 (8.4)	370 (42.7)	<0.001
**Comorbidity, *n* (%)**			
Myocardial infarct	1396 (20.8)	183 (21.1)	0.838
Congestive heart failure	2362 (35.2)	377 (43.5)	<0.001
Peripheral vascular disease	1032 (15.4)	147 (17)	0.229
Dementia	192 (2.9)	31 (3.6)	0.242
COPD	1782 (26.6)	295 (34)	<0.001
Liver disease	710 (10.6)	195 (22.5)	<0.001
Diabetes	630 (9.4)	75 (8.7)	0.48
Renal disease	1661 (24.8)	288 (33.2)	<0.001
**Severe score, median (IQR)**			
SOFA	6 (4,9)	11 (8,14)	<0.001
GCS	13 (10,14)	7 (3,11)	<0.001
APSIII	51 (37,70)	90 (70,109)	<0.001
**Laboratory tests, median (IQR)**			
Lactate (mmol/L)	1.8 (1.3,2.5)	2.2 (1.4,3.7)	<0.001
Glucose (mg/dL)	128.5 (114.6,144.3)	128 (105.2,148.2)	0.02
Creatinine (mg/dL)	1 (0.8,1.6)	1.5 (0.9,2.5)	<0.001
BUN (K/uL)	20 (14,33)	33.5 (20,52.5)	<0.001
Platelets (K/uL)	179 (131,246.5)	176.5 (109.8,259.5)	0.042
Potassium (K/uL)	4.2 (3.9,4.6)	4.3 (3.9,4.9)	<0.001
Sodium (K/uL)	138.5 (136,141)	138.5 (135.5,141.5)	0.903
Bicarbonate (mEq/L)	23 (20.5,25.5)	21.5 (18,25)	<0.001
Calcium (mg/dL)	8.2 (7.8,8.7)	8.2 (7.6,8.7)	0.003
Chloride (mmol/L)	105.5 (101.5,108)	103.5 (99,107.5)	<0.001
HCT (%)	10.4 (1.9)	10.1 (2)	<0.001
PTT (s)	32 (27.9,40.2)	38.1 (30.5,53.5)	<0.001
WBC (K/uL)	11.9 (8.8,15.8)	13.2 (9,18.2)	<0.001
INR	1.3 (1.1,1.5)	1.5 (1.2,2)	<0.001
AG	13.5 (11.5,16)	16 (13.5,19.5)	<0.001
MCH (pg)	30 (28.6,31.3)	30 (28.5,31.5)	0.529
MCHC (g/dL)	32.8 (31.7,33.9)	32.3 (31,33.4)	<0.001
MCV (fL)	91 (87,95)	92 (88,97)	<0.001
RDW (%)	14.9 (13.7,16.4)	16.6 (15,18.3)	<0.001
**Vital signs, median (IQR)**			
Heartrate (min^−1^)	103 (90,118)	113 (97,130)	<0.001
Respiratory rate (min^−1^)	27 (23,32)	30 (25,35)	<0.001
Temperature (°C)	36.3 (35.6,37.3)	36.3 (35.6,37.3)	0.021

**Table 4:** Comparison of basic characteristics of the surviving and dead groups in the Eicu-CRD database.

Variable	Survival	Death	*p* value
**Total**	1402	171	
ICU stay time, days, median (IQR)	4 (2.3,7.4)	4.3 (2.3,8.2)	0.574
Age, year, median (IQR)	66 (54,76)	66 (57,77.5)	0.197
Gender, male, *n* (%)	821 (58.6)	88 (51.5)	0.076
**Medical treatments, *n* (%)**			
Sedatives	321 (22.9)	59 (34.5)	<0.001
Analgesic	162 (11.6)	36 (21.1)	<0.001
Antibiotic	1 (0.1)	0 (0)	0.727
Vasopressin	88 (6.3)	58 (33.9)	<0.001
**Comorbidity, *n* (%)**			
Myocardial infarct	48 (3.4)	9 (5.3)	0.224
Congestive heart failure	101 (7.2)	14 (8.2)	0.641
Peripheral vascular disease	11 (0.8)	1 (0.6)	0.777
Dementia	13 (0.9)	1 (0.6)	0.653
COPD	13 (0.9)	4 (2.3)	0.092
Liver disease	19 (1.4)	3 (1.8)	0.675
Diabetes	11 (0.8)	1 (0.6)	0.777
Renal disease	129 (9.2)	19 (11.1)	0.419
**Severe score, median (IQR)**			
SOFA	7 (5,9)	9 (7,12)	<0.001
GCS	11 (7,14)	9 (6,14)	<0.001
APSIII	55 (41,73)	77 (57,103)	<0.001
**Laboratory tests, median (IQR)**			
Lactate (mmol/L)	1.6 (1.1,2.6)	2.7 (1.5,5.1)	<0.001
Glucose (mg/dl)	124 (105,146)	123 (100,145)	0.531
Creatinine (mg/dl)	1.1 (0.8,1.8)	1.4 (1,2.3)	<0.001
BUN (K/uL)	22 (14,35)	30 (20,48)	<0.001
Platelets (K/ul)	166 (118,234)	171 (91.5,243)	0.416
Potassium (K/ul)	4.1 (3.6,4.5)	4.1 (3.6,4.8)	0.226
Sodium (K/ul)	139 (136,142)	138 (135,142)	0.192
Bicarbonate (meq/L)	23 (20,25)	21 (17,24)	<0.001
Calcium (mg/dl)	8 (7.4,8.6)	8 (7.4,8.4)	0.102
Chloride (mmol/L)	106 (102,110)	105 (100,109)	0.096
HCT (%)	30.7 (26,36)	29.8 (25.8,34.7)	0.188
PTT (s)	32.9 (28,39.4)	35.8 (30.7,45.1)	<0.001
WBC (K/uL)	11.9 (8.2,17)	13 (8.1,18.9)	0.231
INR	1.3 (1.1,1.6)	1.5 (1.2,2.1)	<0.001
AG	11 (8,14)	14 (10,16)	<0.001
MCH (pg)	29.8 (28.1,31.1)	29.4 (27.9,31.1)	0.331
MCHC (g/dL)	32.9 (32,33.8)	32.5 (31.4,33.3)	<0.001
MCV (fL)	90 (86.2,94.4)	91.2 (85.6,96)	0.319
RDW (%)	15.1 (14,17)	16.4 (14.9,18.2)	<0.001
**Vital signs, median (IQR)**			
Heartrate (min^−1^)	92 (79,108.8)	93 (80.5,110)	0.231
Respiratory rate (min^−1^)	19 (15,24)	20 (16,25)	0.014
Temperature (°C)	36.7 (36.3,37.2)	36.5 (36,37)	<0.001

### Feature selection

**Figure 2:**
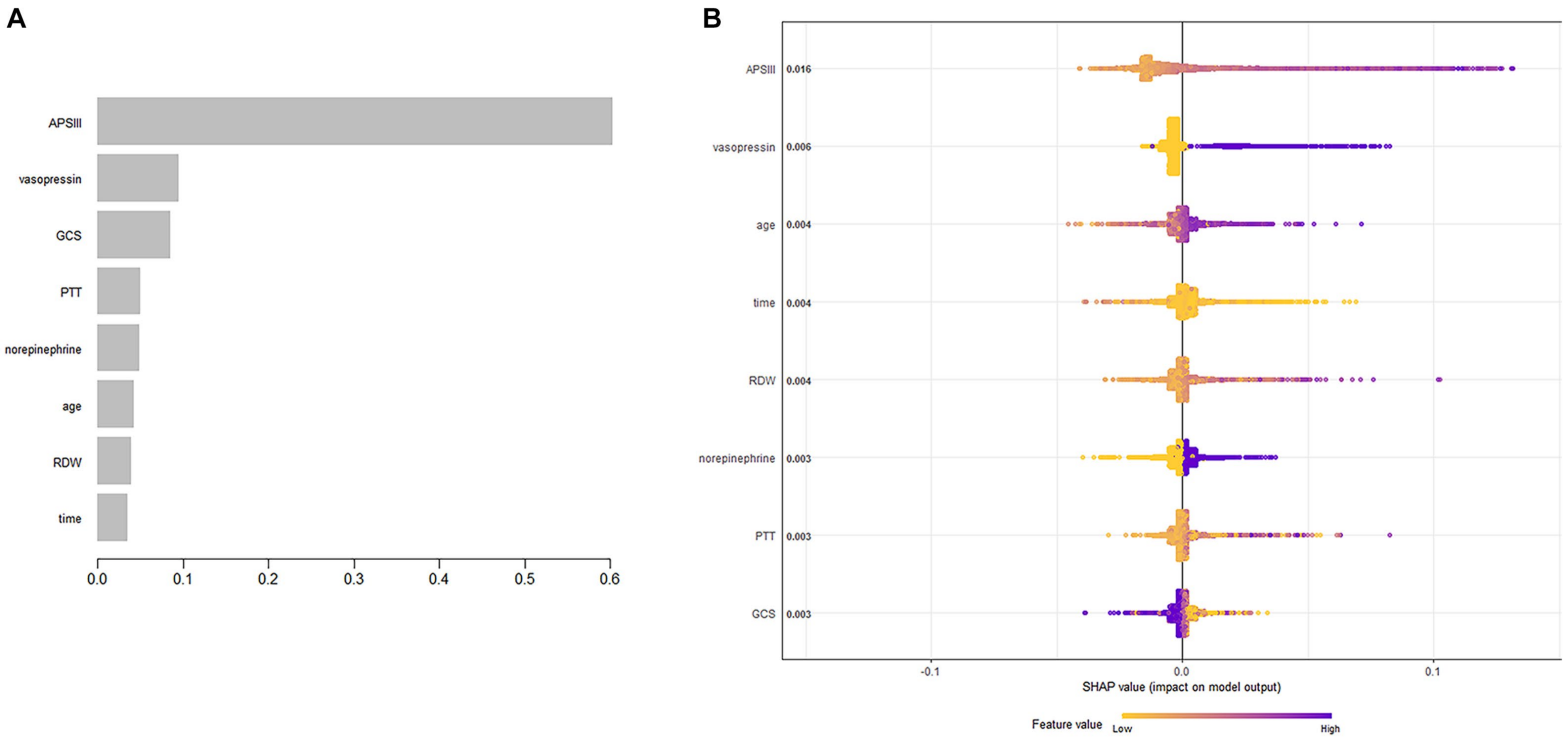
Predictor variables selection. **(A)** Importance of the predictor variables selected by XGBoost. **(B)** The SHAP summary plot.

The XGBoost algorithm identified APSIII, vasopressin, GCS score, PTT, norepinephrine, age, RDW, and length of ICU stay as independent predictors of SAE. Figure 2A presents the importance of each factor influencing SAE. APSIII had the highest score, indicating that determining severity in patients was the most relevant and important factor. Smaller APSIII values indicate a lower output from the model. The GCS score had the smallest effect on the model. Figure 2B presents the SHAP summary plot, which reflects the influence of each factor using the SHAP value in XGBoost and whether they had a positive or negative effect The SHAP plot illustrates the influence of each parameter on the XGBoost model, including the positive (APSIII, vasopressin, age, RDW, PTT, and norepinephrine) and negative (GCS) effects.

**Figure 3:**
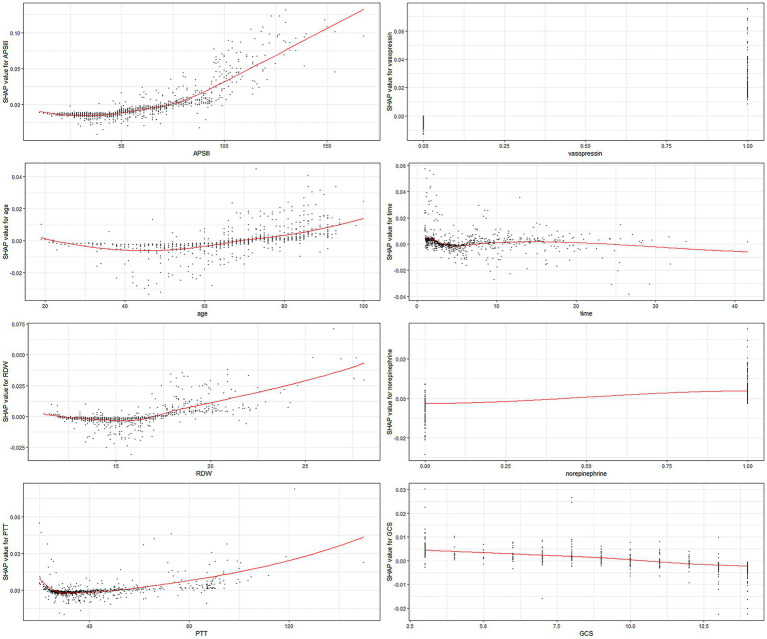
SHAP dependency plot of the XGboost model. The SHAP dependence plot shows how a single feature affects the output of the XGBoost prediction model. SHAP values for specific features exceed zero, representing an increased risk of death. RDW, red blood cell distribution width; PTT, partial thromboplastin time.

Each of the eight factors is represented by a SHAP dependence plot in Figure 3, which illustrates how different characteristics influenced the XGBoost model results. Positive SHAP values for specific factors represent an elevated mortality risk. We found that mortality was correlated with higher APSIII, age, RDW, and PTT, and a lower GCS score. Both longer and shorter ICU stays were associated with lower survival rates. Patients who receive vasopressin and norepinephrine may experience higher mortality rates.

**Figure 4:**
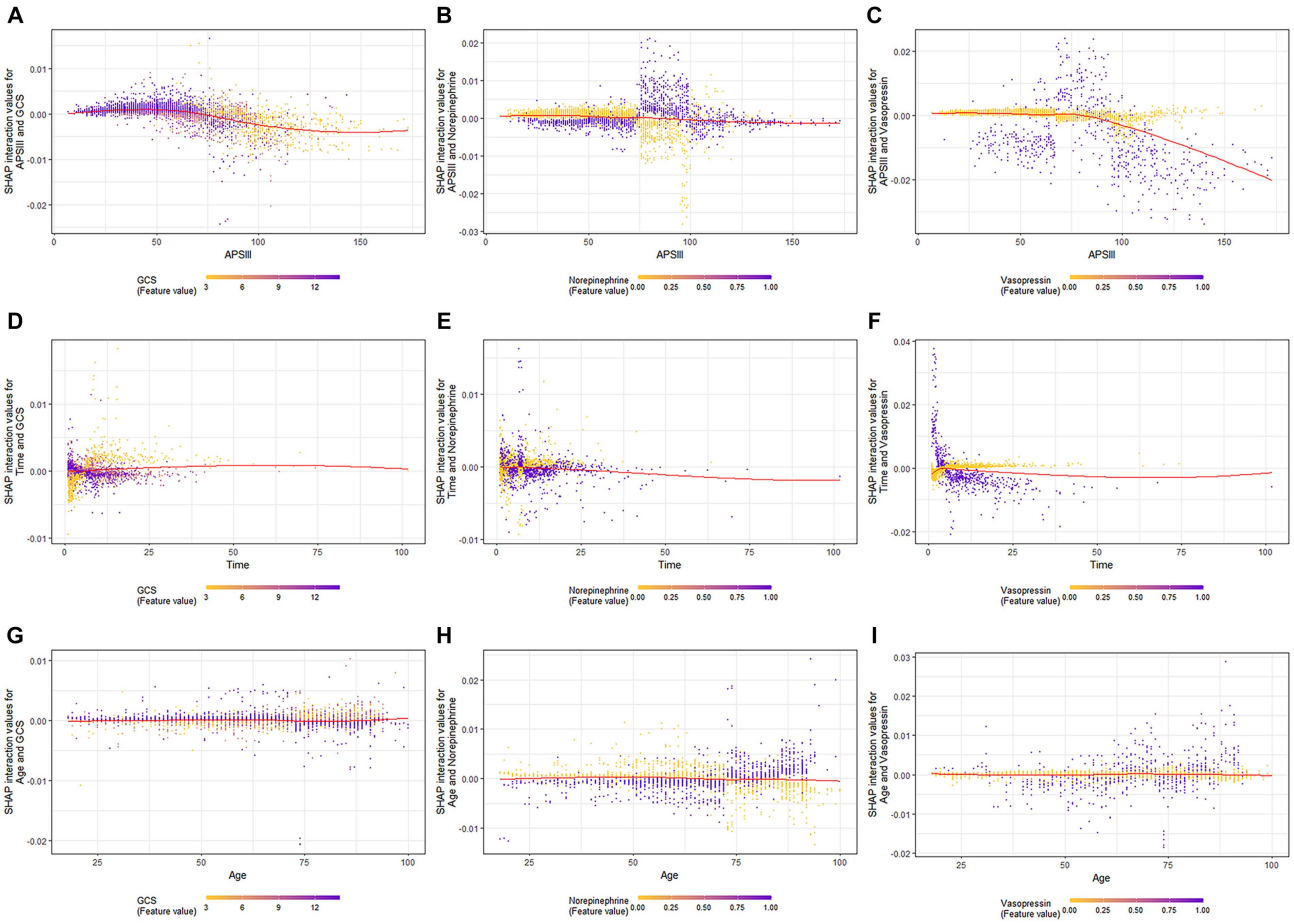
SHAP interaction plot of the eight most essential features for SAE assessment. SHAP, SHapley Additive explanation; XGBoost, eXtreme Gradient Boosting; SAE, sepsis-associated encephalopathy.

When GCS scores were low, the SHAP interaction values of APSIII with the GCS score decreased as APSIII increased (Figure 4A). The interaction effect of APSIII with norepinephrine and vasopressin (Figures 4B,C) did not seem to be affected by differences in norepinephrine or vasopressin use. Samples with the highest SHAP values for death in the ICU were often accompanied by vasopressin use and a shorter ICU stay. When the GCS score was higher, the value of the interaction between time and GCS score decreased as the length of ICU stay increased (Figure 4D). The interaction effect of time in ICU with norepinephrine (Figure 4E) did not appear to be affected by the use of norepinephrine. The value of the interaction between time and vasopressin use decreased as the length of ICU stay increased (Figure 4F). When the GCS score was high, the interaction value between age and GCS score decreased as age increased (Figure 4G), and the interaction value between age and GCS score decreased to a negative value at 73 years old. When norepinephrine was used, the value of the interaction between age and norepinephrine increased with age (Figure 4H) and became positive at 73 years old. The SHAP interaction values for age with vasopressin use also increased with age (Figure 4I).

**Figure 5:**
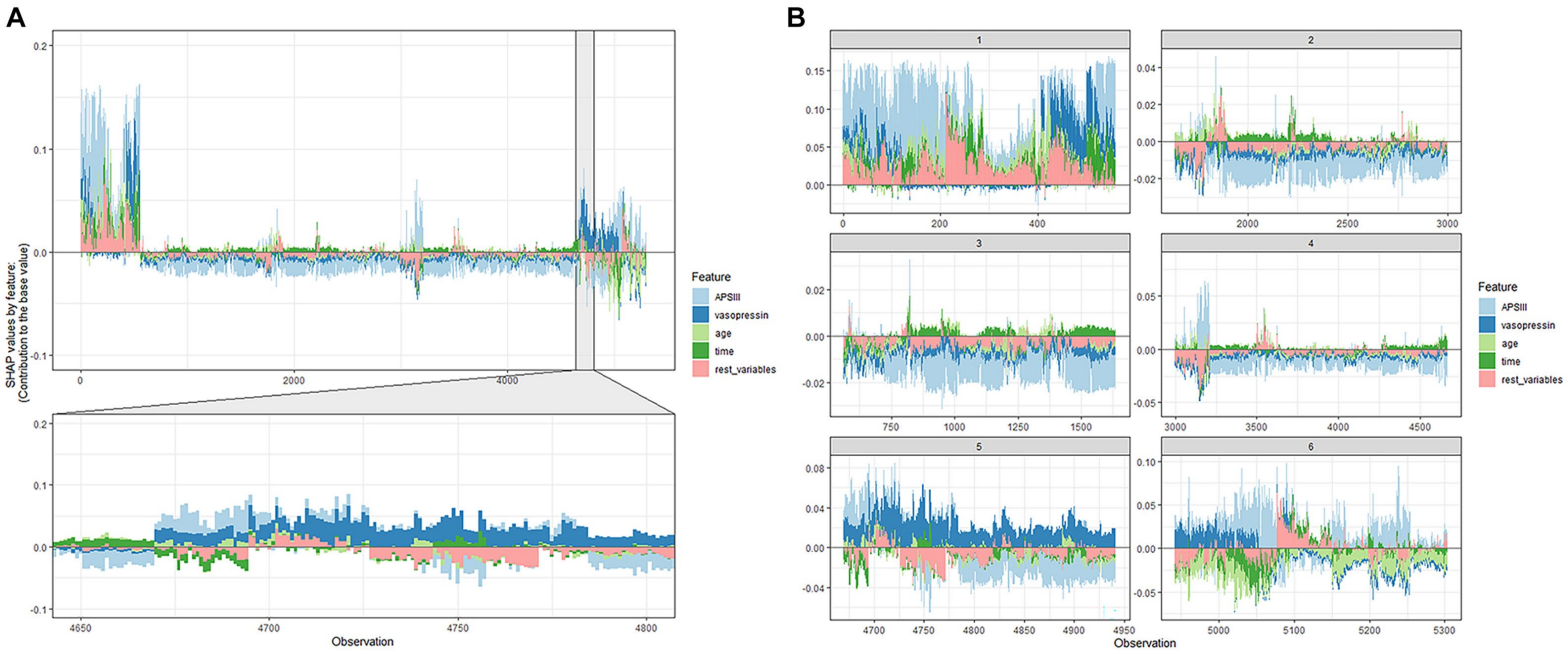
SHAP force plot of the XGboost model. **(A)** Influence plot of macroscopic features for all samples. **(B)** Influence plot of macroscopic features for a random portion of the samples. A positive Shap value represents a positive gain area and a negative Shap value represents a negative gain area.

The ultimate output was obtained as the sum of the attributions from each predictor, as seen in the SHAP force plot (Figure 5), which displays these SHAP values stacked for each observation.

### Discrimination ability

**Figure 6:**
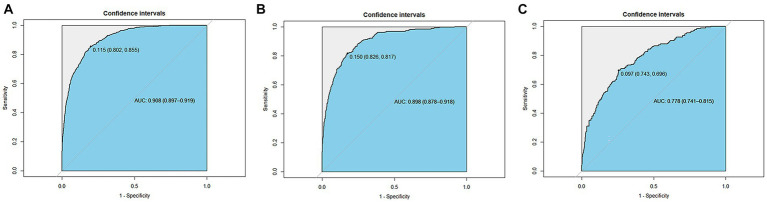
Receiver operating characteristic curves of the XGBoost model. **(A)** The test set (AUC=0.908). **(B)** The internal validation set (AUC=0.898). **(C)** The external validation set (AUC=0.778).

The ROC was used to evaluate the discrimination ability of the model. The XGBoost model test, internal validation, and external validation sets had AUC values of 0.908, 0.898, and 0.778, respectively (Figure 6).

## Discussion

SAE represents a multifaceted encephalopathy, signifying a widespread cerebral impairment stemming from sepsis. It manifests with manifestations such as delirium, coma, cognitive deficits encompassing the loss of learning and memory, and the occurrence of seizures. The pathophysiology of SAE remains partially elucidated. While advances in sepsis research and treatment have lately yielded enhanced prognostic outcomes, the mortality rate of SAE remains disheartening. The identification of risk factors is imperative in grasping the prognosis of SAE.

An XGBoost model was constructed and validated in this study to predict ICU mortality in patients with SAE. The importance analysis of the factors in the XGBoost model suggested that APSIII, vasopressin, age, length of ICU stay, RDW, norepinephrine, PTT, and GCS score are strong predictors of SAE. In this study, the XGBoost prediction model was obtained from the AUC results and had good predictive power. SHAP also offered credible visual interpretation of the predictions, encompassing both positive and negative impacts. In this study we not only calculated values of general parameters for predicting the probability of death in the ICU, but also presented a visual explanation for specific patients using SHAP plots. The predictive value of a clinical factor for the XGBoost model increased with the average absolute SHAP value of each factor. Each factor was averaged to provide a homogeneous perspective, and the interpretation of SHAP was based on each individual patient (17). SHAP has two advantages: (1) it considers the effects of individual factors and the synergy between factors, which can solve the multicollinearity problem, and (2) SHAP determines whether the influence is favorable (18).

In the current investigation, APSIII emerged as the most weighty contributor in the importance plot, underscoring its robust capacity to predict mortality in the ICU for individuals grappling with SAE. As an integral facet of the APACHE system, APSIII aptly showcases its aptitude in prognosticating the mortality rates of patients grappling with severe sepsis and septic shock (19). In a large number of studies it has been found that survivors have significantly lower APACHE III scores than deceased patients and that higher scores (OR 1.11,95% CI 1.05–1.18, *p* = 0.001) are associated with increased in-hospital all-cause mortality in patients with severe sepsis (20, 21). Inflammatory response, immunosuppression, and multiple organ dysfunction syndrome may be responsible for the high scores in patients with SAE (1, 22). A predominant clinical characteristic of SAE is the alteration in the level of consciousness. In milder cases, there is a reduction in attention and alertness, accompanied by symptoms like anxiety and delirium. In severe instances, it may lead to stupor or coma. Long-term cognitive impairments encompass deficits in memory, attention, verbal fluency, and executive functions, significantly impacting the quality of life for survivors. In a study concentrated on discerning initial and potentially amendable factors of SAE upon admission to the ICU, it was determined that even slight alterations in cognitive function, as defined by a GCS score of 13–14, were autonomously correlated with mortality at the point of ICU admission (10, 23). Furthermore, our findings confirm the independent role of the GCS score as a risk factor for ICU mortality in SAE patients. This reinforces the utility of the GCS score and APSIII in gauging the severity and prognosis of individuals afflicted with SAE.

Norepinephrine and vasopressin are now commonly used in clinics as vasoactive drugs. In the Surviving Sepsis Campaign guidelines, norepinephrine is recommended as the vasopressor for sepsis treatment (24), and often vasopressin is used as an adjunct to sepsis. Maheshwari et al. found that the significant blood pressure response to VAS was substantially linked to reduced survival probability in patients with septic shock (25). However, there are only treatment guidelines for sepsis and septic shock (15, 17), with a lack of specific treatment guidelines for SAE (24, 26). To realistically assess ICU mortality in SAE, clinicians should be aware of other treatment options for SAE in order to improve the corresponding survival assessment system. Currently, no specific therapeutic interventions are tailored for SAE. Treatment protocols are established on the comprehensive management of sepsis, with a predominant focus on symptoms associated with cerebral maladies, while endeavoring to minimize detriment to the central nervous system. Early-stage resuscitation is acknowledged as a pivotal therapeutic strategy for sepsis, and the administration of vasoactive agents correlated with normal arterial pressure subsequent to initial fluid therapy can mitigate the severity of sepsis (27). Furthermore, glucocorticoids, alternative markers, and modulators of the neuroimmune axis have been under consideration for addressing sepsis-induced cognitive impairments (28, 29). Indoleamine 2,3-dioxygenase, impacting the inflammatory cascade, is identified as a potential therapeutic target for central nervous system disorders, fostering cognitive enhancement in sepsis patients (30).

Most of the patients with SAE in this study stayed in the ICU for less than 1 week, and the length of ICU stay had an overall negative effect on the outcome. Related studies have found length of ICU stay to be related to disease severity (31), which has important implications for the wise use of scarce medical resources (32). Elderly patients with SAE admitted to the ICU mostly died earlier than did younger patients with SAE, which is supported by the findings of Martin et al. (33). The introduction of comorbidities harm immune function as age progresses, which causes patients with critical illness to deteriorate more rapidly. Geriatric patients may be more vulnerable to CNS issues, particularly if hypertension, diabetes mellitus, or acute renal injury is the underlying illness (10, 34, 35). Older hospitalized people need more-specialized nursing or rehabilitation care. These findings offer guidance on how to allocate healthcare resources for patients with SAE and offer suggestions for future research projects and patient interventions.

RDW may be therapeutically valuable for predicting the future course and prognosis of various disorders, including stroke, atrial fibrillation (36), COPD (37), community-acquired pneumonia, and sepsis (38). Currently, several studies have indicated that RDW possesses not only diagnostic significance but also serves as a prognostic factor for sepsis, signifying systemic dysfunction and immune response dysregulation. Víctor Moreno-Torres, MD, et al. have proposed that RDW enhances the discriminative capacity of SOFA, LODS, APACHE-II, and SAPS-II, rendering it a potential parameter within these prognostic scoring systems (39). A study by Sadaka et al., which included 279 patients with septic shock, suggested that elevated RDW at admission was related to death in the ICU in both adults and neonates (40). This finding was also demonstrated in another study, which found that the addition of RDW to the ICU scoring system improved its mortality predictions (41, 42). The present study indicated there was a higher risk of dying from SAE in the ICU when RDW levels were high. Elevated RDW reflects a severe dysregulation of red blood cell homeostasis, which may be an important prognostic factor for SAE. The mechanistic relationship between RDW and the ICU mortality rate in SAE remains obscure. However, research suggests that oxidative stress may contribute to the detrimental impact of RDW on the prognosis of SAE, as oxidative stress levels exhibit a positive correlation with RDW (43). Apart from this, the inflammatory response in septic patients shortens red blood cell lifespan, impairs red blood cell maturation, resulting in premature release, and thus elevating RDW (44, 45). Furthermore, proinflammatory cytokines inhibit erythropoietin-induced red blood cell proliferation and maturation, also leading to an increase in RDW (46). This may represent another rationale for the association between RDW and ICU mortality.

One strength of this study was the external validation of the SAE mortality risk model using the eICU-CRD database, which confirmed its efficacy. SHAP allows visualization of XGBoost models, and its sound visual interpretation greatly increases the confidence that clinicians have in the application of machine learning. However, there were some limitations to this study. First, only data from the US were utilized to construct and validate the model, which might reduce its applicability to other regions of the world. Furthermore, in retrospective studies, it is inevitable to relinquish certain variables with a substantial amount of missing values. Various unmeasured confounding variables such as race and treatment modalities, along with inflammation-related data, could potentially influence the mortality risk of SAE patients. Therefore, given the constraints of the MIMIC-IV database and the eICU-CRD database, it is plausible that the XGBoost model may have omitted certain pivotal factors. Finally, the divergence in the origins of these two patient cohorts, hailing from distinct databases, has led to disparate study timelines between the training and external validation cohorts. This temporal incongruity might be a pivotal element contributing to the attenuation of this model’s efficacy within the eICU-CRD database.

## Conclusion

This study validated the efficacy of machine-learning-based XGBoost for early outcome predictions for patients with SAE. The SHAP method improves the readability of XGBoost models and aids doctors in comprehending the logic behind findings obtained from such models.

## Data availability statement

The original contributions presented in the study are included in the article/supplementary material, further inquiries can be directed to the corresponding author.

## Ethics statement

The article does not belong to the scope of the Ethics Committee Review and does not need to be reviewed according to the current ethical standards. Both MIMIC-IV and ICU-CRD are public databases and are public resources. The authors have passed the application of both MIMIC-IV and ICU-CRD databases, completed the CITI examination and account registration, and have the permission to use them.

## Author contributions

JG: Validation, Writing – original draft, Writing – review & editing. HC: Writing – original draft, Writing – review & editing. ZW: Methodology, Writing – review & editing. MQ: Validation, Writing – review & editing. JiL: Conceptualization, Writing – review & editing. JuL: Funding acquisition, Writing – review & editing.
